# The Emergence and Development of Animal Research Ethics: A Review with a Focus on Nonhuman Primates

**DOI:** 10.1007/s11948-020-00219-z

**Published:** 2020-04-29

**Authors:** Gardar Arnason

**Affiliations:** Institute of Ethics and History of Medicine, University of Tübingen, Gartenstrasse 47, 72074 Tübingen, Germany

**Keywords:** Research ethics, Nonhuman primates, Animal research, Animal research ethics

## Abstract

The ethics of using nonhuman animals in biomedical research is usually seen as a subfield of animal ethics. In recent years, however, the ethics of animal research has increasingly become a subfield within research ethics under the term “animal research ethics”. Consequently, ethical issues have become prominent that are familiar in the context of human research ethics, such as autonomy or self-determination, harms and benefits, justice, and vulnerability. After a brief overview of the development of the field and a discussion of relevant theoretical ethical frameworks, I consider two of these issues, namely autonomy and self-determination on the one hand, and harms and benefits on the other hand. My concern is with philosophical and ethical issues, rather than animal research oversight. I focus my discussion on nonhuman primates, as the most plausible nonhuman candidates for this approach. I conclude that the approach, although promising, depends strongly on the moral status of nonhuman research subjects.

## Introduction

Within the field of philosophical ethics, the ethics of using nonhuman animals in biomedical research is usually seen as a subfield of animal ethics. In that context the central problem is the general justifiability of nonhuman animal research. In contrast to the majority of public views and public policies, most major works in animal ethics have been strongly opposed to the current use of nonhuman animals for research, varying from arguing for restrictive positions to advocating abolition.^1^ In recent years, however, problems in the ethics of animal research have increasingly been discussed within a framework similar to human research ethics, sometimes under the term “animal research ethics” (Gilbert 2012; Beauchamp et al. 2014; DeGrazia and Beauchamp 2015).

When the ethics of animal research is placed in the context of research ethics, questions and problems become prominent that are familiar in the context of human research ethics but often less so in the context of animal ethics; and those problems that are prominent in animal ethics, such as animal harm, are to some extent approached differently. Four of the main issues that come to the fore in animal research ethics are autonomy or self-determination, harms and benefits, justice, and vulnerability (Ferdowsian and Choe 2013). When considering these four issues, it may seem that autonomy is an issue that is central to human research ethics but not applicable to animal research ethics, while the issue of harms and benefits is already a central issue in animal ethics, and hence not novel in the context of animal research ethics. I will therefore pay particular attention to these two issues.

The question of the general justifiability of animal research barely matters in animal research ethics, but that does not mean that animal research ethics supports the status quo in animal research or even that it is more permissive than most animal rights positions. On the contrary, the growing literature in animal research ethics tends to be very critical of current practices in nonhuman animal research, but its concerns are much wider and more differentiated than merely the question whether nonhuman animal research can be justified or must be abolished.

This is not to say that animal research ethics, in the sense described here, is fully independent of animal ethics. Many of the concerns that give rise to discussions about animal self-determination and agency, harms and benefits, justice, and vulnerability, are motivated by and seek support from classical works in animal ethics. Scientific research on animal cognition and behavior has also influenced and driven debates both within animal ethics about the justification of using animals in biomedical research and discussions in animal research ethics about specific problems and principles in animal research. The two fields are closely connected.

The extent to which human research ethics concepts and concerns apply to nonhuman animals is closely related to the animals’ moral status. At the extremes, if sentient nonhuman animals (animals that can feel pain) have the same moral status as paradigmatic humans, namely personhood or full moral status, the concepts and concerns of human research ethics will apply to them in the same way as to humans; if sentient nonhuman animals have no moral status and do not matter morally at all, neither the concepts nor concerns of human research ethics will apply to them. If nonhuman animals are considered to have a moral status, but lower than humans, then the consequences for the applicability of both concepts and concerns need to be worked out. By moral status I mean the extent to which something matters morally for its own sake. There is no agreement on what features determine moral status, but according to many accounts moral status depends significantly on cognitive capacities and sentience (Jaworska and Tannenbaum 2018). Nonhuman primates, and in particular nonhuman great apes, are in evolutionary terms our closest relatives. They have the highest cognitive capacities of all nonhuman animals and therefore, presumably, a greater capacity for suffering. Some species of nonhuman primates may even have some features of personhood.^2^ If the concerns of human research ethics apply to nonhuman animals at all, they will apply to the greatest extent to nonhuman primates. This makes nonhuman primates an interesting test-case for animal research ethics.

As animal research ethics establishes itself as a subfield of research ethics and the literature on various topics in animal research ethics grows, it is useful to reflect on the development of the field and what it means for the ethics of animal research to be placed in the context of research ethics. In what follows I will attempt to describe the current state of animal research ethics with a focus on nonhuman primates as subjects of biomedical research. After a brief overview of the development of the field and a discussion of relevant theoretical ethical frameworks, I consider two of the main issues that, perhaps unexpectedly, set animal research ethics apart from the traditional animal ethics approach, namely agency and self-determination on the one hand, and harms and benefits on the other hand.

My aim here is to review and reflect on the discussion of the ethics of animal research within the field of research ethics, with nonhuman primates as my main example and a focus on the two issues of agency/self-determination, and harms and benefits. I make three central claims regarding animal research ethics: (1) the ethics of animal research is increasingly being discussed in the context of research ethics; (2) this has led to a richer and more fruitful discourse on animal research, as is exemplified by the issues of agency/self-determination and benefits/harms; and (3) the moral status of nonhuman animals is fundamental to the applicability of the research ethics framework to the ethics of animal research. I hope to advance the discussion of the use of nonhuman sentient animals, in particular nonhuman primates, in biomedical research, by providing an overview of how the debate is increasingly taking place within the framework of research ethics, and what the assumptions and implications of using that framework are. My aim is not to argue for or against the use of animals in research, but I do assume that some animal research can be justified, at the very least if it is conducted under the same ethical restrictions as research on humans. My focus is on philosophical issues in animal research ethics, but I am aware that the term “animal research ethics” is also used to refer to animal research oversight and regulatory frameworks, including laws and regulations, professional codes, ethical guidelines, and institutional rules and procedures. A parallel division exists in the field of human research ethics.

## The Emergence of Animal Research Ethics

In his contribution to *The Routledge Companion to Bioethics*, Tom L. Beauchamp (2014, p. 262) calls animal research ethics “a recently coined term”. It is, indeed, only in the last decade, that animal research has been discussed extensively within the framework of philosophical research ethics, but the term “animal research ethics” goes back at least to the 1980s.^3^ Jerrold Tannenbaum and Andrew N. Rowan conclude in an article published in 1985 that “animal research ethics is still at a rudimentary state of development” (Tannenbaum and Rowan 1985, p. 42) and compare it to the state of human research ethics in 1966 when Henry Beecher published his influential article on the ethics of biomedical research, revealing 22 examples of research protocols where human subjects were treated unethically (Beecher 1966). I note that the lesson drawn from Beecher’s terrifying list of human rights abuses in biomedical research was not that human biomedical research should be abolished, but that the conditions for justifiable research had to be clarified within a better research ethics. Similarly, examples of animal abuse in biomedical research should not lead to calls to abolish animal research, but to improvements in animal research ethics, in regulation and practice. Discussing the use of animals in biomedical research within the context of research ethics signifies exactly such a change of emphasis. It moves the focus of the discussion away from the question of the justifiability of animal research and places a stronger emphasis on the ethical frameworks within which it should be regulated and practiced.

Like Tannenbaum and Rowan before them, Hope R. Ferdowsian and John P. Gluck also discuss Beecher’s work in a recent article and claim that “that there are significant parallels between Beecher’s observations about human research in 1966 and contemporary problems with animal research” (Ferdowsian and Gluck 2015, p. 391). One might get the impression that not much has happened in animal research ethics during the almost 30 years between the publication dates of these two articles, except that examples of ethically questionable animal research keep accumulating. That impression is not accurate. Since the 1980s, animal research proposals must increasingly be approved by animal ethics committees, which may be institutional, regional or national. In the U.S., federally funded animal research has to be reviewed by an institutional animal care and use committee (IACUC). It is parallel to institutional review boards (IRBs) in human research. In Europe, recent legislation on animal research, Directive 2010/63/EU on the protection of animals used for scientific purposes, stipulates that animal research must receive ethical approval by a competent authority, meeting a specific list of ethical and scientific requirements (European Parliament and the Council of the European Union 2010). The Directive also puts the use of nonhuman primates into the spotlight. The former Directive it replaces had only one paragraph mentioning nonhuman primates (European Parliament and the Council of the European Union 1986), but the current Directive refers 48 times to them, including three preambles devoted solely to nonhuman primates. The 3R framework proposed by Russell and Burch in 1959 is increasingly being applied, requiring scientists to *replace* animals with alternative methods (or lower species) if possible, to *reduce* the number of animals to the minimum required for statistically valid results, and to *refine* the use of animals by minimizing their pain and suffering as well as improving husbandry, housing, and welfare (Russell and Burch 1959). All three requirements can be recognized in Directive 2010/63/EU and corresponding national legislation in Europe. The directive also prohibits the use of great apes except for very specific circumstances, but great apes have not been used for research in Europe since 1999 (UK Home Office 2014, p. 23). In the United States, the use of chimpanzees is being phased out by the National Institutes of Health, effectively ending the use of great apes in research in the U.S. (Beauchamp et al. 2014; Reardon 2015). These developments undermine the claim that the situation in animal research is comparable to the one in research involving human beings in the 1960s.

In the academic literature, one possible starting point for the emergence of animal research ethics is the publication in 2006 of a special issue of *Theoretical Medicine and Bioethics* focusing on animals (DeGrazia 2006). This special issue includes two articles that draw a comparison between the use of humans and the use of animals in biomedical research (Walker 2006; Pluhar 2006). In 2012 the Hastings Center published a Special Report entitled “Animal Research Ethics: Evolving Views and Practices” (Gilbert et al. 2012). The report includes a section about chimpanzee research in the United States, but it does not explicitly place the issues in the context of research ethics. The report was followed by another special issue of *Theoretical Medicine and Bioethics* in 2014, which is primarily concerned with chimpanzee research in the United States (Beauchamp et al. 2014). Here questions about autonomy, consent, vulnerability, and harm are directly addressed. In 2015 *Cambridge Quarterly of Healthcare Ethics* published a special section entitled “Moving Forward in Animal Research Ethics” (DeGrazia and Beauchamp 2015) and a year earlier the same journal published a special section on “Neuroethics and Animals” (Buller et al. 2014). Both special sections contain articles that discuss the question of autonomy of research animals in terms of the possibility to assent or dissent (Fenton 2014; Kantin and Wendler 2015). The impetus for this discussion about animal autonomy came from a US Institute of Medicine report on the use of chimpanzees for biomedical and behavioral research, released in 2011, which led to the discontinuation of chimpanzee research in the U.S. (IOM 2011). I will consider the issue of autonomy in greater detail below.

Although the literature on animal research in the context of research ethics has been growing rapidly, it does not mean that animal research is no longer discussed in the context of animal ethics. Scholarly articles and books discussing animal research in the familiar animal ethics context, mostly calling for the abolition of animal research (e.g., Herrmann and Jayne 2019), continue to be published. Those who have published on animal research in the research ethics context, or those who use the human research ethics framework to some extent when discussing animal research, may not consider themselves moving away from animal ethics. It is nonetheless possible to discern a growing interest in the framework of human research ethics, which has given rise to a body of work large enough and with enough in common to describe it as a distinct field of research, regardless of the intention of the individual authors who have or are contributing to it. One of the main features of animal research ethics, as a subfield of research ethics, is in the ethical approach, with less emphasis on the standard utilitarian (e.g., Singer) or deontological (e.g., Regan) ethical frameworks to an increasing interest in the principlism of Beauchamp and Childress (2013).^4^ To draw out this difference, let me take a closer look at the ethical frameworks that are usually applied to the issue of animal research.

## Ethical Frameworks for Animal Research Ethics

An overview of the main ethical positions concerning the use of nonhuman animals in research reveals a variety of conflicting positions even within the same general theoretical approach. A characteristic trait of medical ethics can also be observed in animal ethics: the limited direct benefit of the “classical” moral theories. No general theoretical approach in ethics can be applied directly and conclusively to determine the moral status of animals, the justifiability of animal research, nor what sort of animal research ethics is appropriate. That is, defendable positions for abolishing animal research, for restricting animal research, and for the current practice, can and have been developed within each of the general theoretical approaches. In this section I will briefly consider some of the main approaches to animal ethics and what they mean for animal research ethics.

Utilitarian arguments have been applied to support a near abolitionist position about animal research in general (Singer 1975), but also positions in favor of animal research generally (Brody 2012; Cohen 1990; Frey 1997) and a position in favor of nonhuman primate research specifically (Weatherall 2006). The best known utilitarian position in animal ethics is Peter Singer’s preference utilitarianism as developed in his books *Animal Liberation* (Singer 1975) and *Practical Ethics* (Singer 1979). According to that position, we should do what best satisfies our well-considered preferences or objective interests, which in the case of animals amounts to matters of pleasure and pain. Singer also emphasizes the importance of the principle of “equal consideration of interests”. Singer’s position does not rule out animal research, provided its utility (in terms of satisfying interests) is greater than the harm caused. This, Singer claims, is very rarely the case in animal research (Singer 1975).

Current regulatory frameworks for animal research are almost exclusively utilitarian in nature. They aim to minimize the suffering of animals and, in Europe at least, the suffering has to be justified by the potential scientific or medical benefits of the research. This is in stark contrast to human research ethics, where deontological concerns dominate. To use a well-known phrase from Robert Nozick, we have “utilitarianism for animals, Kantianism for people” (Nozick 1974, p. 39).

Although Singer’s utilitarian animal ethics has been very influential, animal ethicists and animal advocates have overwhelmingly relied on deontological approaches, in particular animal rights, when arguing against animal research. The animal rights approach has produced some of the strongest positions against the use of animals in research, for example in Tom Regan’s work (Regan 2004 [1983]) and more recently in the Kantian ethics of Christine Korsgaard (2018). Some animal rights advocates argue against the use of animals for any human purpose (Francione 2009). On the other hand, the classical Kantian deontological approach recognizes only limited, indirect duties towards animals, and some social contract theories put animals squarely outside the moral realm altogether, allowing at most that we have “duties of compassion and humanity” to animals (Rawls 1999, p. 448). Rather than focusing on rights and duties, some deontological theories are based on the concepts of dignity and justice, such as Nussbaum’s capabilities approach to animal ethics (Nussbaum 2004). Nussbaum’s theory would vastly limit human uses of animals, but with the notable exception of allowing the use of animals for biomedical research.

Just as arguments can be made for a wide range of positions on the use of animals for biomedical research within both utilitarian and deontological moral frameworks, virtue ethics provides little guidance in itself. Rosalind Hursthouse (2006), for example, argues that what we can and cannot do with nonhuman animals depends on the circumstances and our relationship to the animals. The basic moral prescription is to act virtuously, in this case to treat animals with compassion and justice. She assumes that animal research is mostly (but not necessarily always) cruel and useless, and that the just and courageous individual should support actions and organizations that aim to stop cruel and useless animal research. Garret Merriam (2012) similarly argues that from the standpoint of virtue ethics much of animal research is unjustified, very little of it is clearly justified, and the rest is in between, requiring careful moral judgment. Walker (2018) has applied virtue ethics specifically to nonhuman primate research, focusing on the virtues and character development of primate researchers and the social and institutional structures involved in the development of the moral character of researchers. These virtue ethics approaches promote moral reflection, the development of moral character of those involved in animal research, and the construction of caring relationships between researchers and their animal subjects, but none of them suggests an absolutist position with regard to the practice of animal research.

Some of the most militant opposition to the use of animals in research is skeptical or even openly hostile to any theoretical basis for its views. This opposition appeals to persons’ empathy with animals and applies a strategy of “exposure and persuasion.” The starting point is that animals are brutally abused and exploited and that bringing animal suffering and injustice into the public eye is the most effective strategy to persuade people to oppose the use of animals in research or in other areas (Aaltola 2011). On this view, any theoretical approach just gets in the way or distracts from the political-activist agenda. Organizations supporting the use of animals in research often appeal to the benefits of animal research for children and pets, rather than engage in philosophical debates, but I am not aware of any academic discussion by supporters of animal research about the usefulness of a comparable anti-theoretical position in favor of animal research.

As the discussion of ethical issues in animal research in the context of research ethics has been growing, the animal ethics discussion has independently been moving from applied ethics to political theory in what has been termed “the political turn”. Much of that discussion is concerned with issues of justice and rights, viewing at least some animals as members of our societies and examining the implications of that (Donaldson and Kymlicka 2011; Garner and O’Sullivan 2016; Cochrane 2018). The political turn in animal ethics has significant implications for animal research ethics, in particular if animals are accorded the same basic rights as humans. This would not result in the abolition of animal research, but it would put severe limits on it, requiring animal research to adopt research ethics that are very similar to current human research ethics (Arnason 2017, 2018a).

This brief survey of how major ethical frameworks have been applied to animal research suggests that broad moral theories do not provide clear answers or a single approach to the question of the justification of animal research. Rather than looking for general answers from moral theories about whether animal research is generally justifiable, recent work on animal research ethics has considered topics that are closer to human research ethics than animal ethics. This work includes, to give a few examples, Walker (2006), Pluhar (2006), and Ferdowsian and Gluck (2015) comparing the ethics of human and nonhuman research, Fenton (2014), Beauchamp and Wobber (2014), and Wendler (2014) on consent and autonomy of nonhuman primates, Kantin and Wendler (2015) on assent and dissent in animal research, Johnson and Barnard (2014) on chimpanzees as vulnerable subjects, Ferdowsian and Fuentes (2014) and Beauchamp and Morton (2015) on harms and benefits in human and nonhuman research, and Choe Smith (2014) on justice in subject selection in animal research. In the following sections, I will focus on two of these topics, autonomy and harm. They are of particular interest for almost opposite reasons: autonomy may seem to be a topic in human research ethics that does not apply in animal research ethics and harm may seem to be a topic that has already been extensively discussed in animal ethics.

## Autonomy, Self-Determination, and Agency

Respect for autonomy, or the person, has been one of the cornerstones of research ethics at least since the publication of the Belmont Report (National Commission 1979). The principle can be found even earlier, in terms of respecting the voluntariness of the subject, in the publication of the Nuremberg Code in 1949. The very first sentence of the Nuremberg Code states simply: “The voluntary consent of the human subject is absolutely essential” (Shuster 1997). It ensures that the potential research subject can protect her own interests, by making an informed and voluntary decision about participating in research. It also ensures respect for autonomy and respect for the person.

It is obvious that nonhuman primates and nonhuman animals generally cannot give informed consent for their participation in research, but it is far from obvious what follows from that fact. One might insist that if a research subject cannot give informed consent, whether the subject is human or not, then she cannot be used in research at all. This position requires a complete abolition of animal research, since no nonhuman animal could be used ethically in research if informed consent is an absolute requirement. When one considers the justification of the requirement for informed consent, this position quickly collapses. If the justification is based on respect for autonomy or respect for persons, then the requirement does not hold in the case of nonhuman animals, since they are neither autonomous nor persons (with the possible exception of great apes), so there is neither autonomy nor personhood to be respected.^5^ If the justification of the requirement for informed consent is to protect the interests of research subjects, then one might simply seek other ways to protect the interests of subjects who are not able to give informed consent. In either case, the requirement for respect of autonomy or the person in the form of informed consent is not only impossible but also irrelevant for nonhuman animal research (Weatherall 2006, p. 129).

Even for nonhuman primates that cannot be considered autonomous, one might still argue that we should consider some sort of a parallel to respect for autonomy, such as respect for agency or self-determination. There is hardly any doubt that nonhuman primates, and other animals, have volition and preferences, and act on those, although they can reflect neither on their preferences nor the reasons they may have for or against acting on them. In that sense they are agents even if they are not autonomous. Such agency, or self-determination, may require that we take it into our moral consideration. The justification of such a requirement could be that they can protect their own welfare interests, to some extent at least, by avoiding activities that cause them pain or distress. Following the terminology of Kantin and Wendler (2015), we can term these welfare-based reasons. One might also attempt to justify a principle of respect for animal agency or self-determination by arguing that agency or self-determination is itself morally relevant independently of welfare. These can be termed agency-based reasons. In this case, the nonhuman animal in question would have a morally relevant interest in exercising its capacity of agency or self-determination, as opposed to merely having an interest in avoiding suffering. The difficult theoretical question here is whether agency, or self-determination, has any moral relevance (or even meaning) in the absence of self-consciousness or the ability to reflect on one’s actions, reasons, values, and beliefs, that is, the sort of features that are usually thought to constitute autonomy. I will not argue for an answer to that question, neither affirmative nor negative, but I do suggest that agency or self-determination is morally relevant at least for welfare-based reasons and as such is a parallel in animal research ethics to the principle of respect for autonomy in human research ethics.

If we accept a principle of respect for agency with regard to research on nonhuman animals, the question remains what form the principle would take in practice. Nonhuman animals cannot consent nor even assent, in the sense that children may be able to assent to research when they are not competent to give informed consent (Fenton 2014, pp. 133–134). Assent requires some grasp of what is going on or going to happen during a specific procedure, which is outside the cognitive capacity even of nonhuman primates (Diekema 2006, p. S9).^6^ Dissent may be possible; however, since it does not require any information about procedures or actions, it consists simply in a wish to be removed from a painful or stressful situation. The opposite of dissent, in this sense, is acquiescence rather than assent. A principle of respect for agency could then take the form of requiring acquiescence from the nonhuman animal during research and conversely respecting animal dissent (Fenton 2014). Such a requirement would clearly put severe limits on nonhuman animal research.

A related but less demanding requirement might consist in seeking voluntary participation from nonhuman animals, in particular nonhuman primates, for research. This is clearly a lower requirement than assent, because no understanding of the procedures and their purpose is required of the research subject. It is similar to acquiescence, but can be considered less demanding as it is induced through training which itself may be considerably more coercive. This sort of voluntariness is increasingly required in some nonhuman primate research, where the primates are trained through positive reinforcement to do such things as offer their arm for injections or the taking of blood samples, entering a primate chair, and performing various actions or solving tasks for rewards. The reasons for respecting voluntariness are in part welfare-based, as applying physical force may cause pain or distress, and in part they are practical, where applying force is not effective. Some common research procedures, for example in primate neuroscience, require the voluntary participation of the primates, since they cannot be physically forced to solve tasks, perform complex actions or make decisions. If they suffer from pain, fear or distress, they might either stop their activities or lose their concentration or interest with the result that the data obtained would be useless. One objection to the practice of seeking voluntariness is that it is mere manipulation and without any moral benefit (Beauchamp and Wobber 2014). This may be the case when one considers agency based reasons, but in so far as the practice of seeking voluntariness reduces suffering, it has a moral benefit for welfare-based reasons.

I hope to have shown in this section that although respect for autonomy does not apply to nonhuman primates (except perhaps great apes), animal agency can have moral significance, at the very least for welfare-based reasons, and such concerns can be compared to the principle of respect for autonomy in human research ethics. Although animals cannot give informed consent, they can often communicate their preferences and express their dissent (or acquiescence) to participating in a research procedure. Nonhuman animals are unlikely to be able to assent to anything, with the possible exception of chimpanzees and other great apes. The moral importance of nonhuman dissent depends in the end strongly on the moral status of the dissenting nonhuman animal and in particular on whether we can be ethically justified in sacrificing its welfare for human benefits.

## Harms and Benefits

Human research ethics limits in practice the level of harm or risk that human research subjects can be exposed to, even if there are research subjects willing to give informed consent to harmful research. Research ethics codes and regulations generally do not specify any limits to risks or harms to human participants, but rather require that risks are minimized (WMA 2013, art. 17; CIOMS 2016, pp. 2, 9–13; US Department of Health and Human Services 2018, §46.111(a)(1)), that benefits outweigh risks (WMA 2013, art. 16; CIOMS 2016, pp. 9–13; US Department of Health and Human Services 2018, §46.111(a)(2)), and, in the case of the Declaration of Helsinki, that the welfare, rights, and interests of the research subject have priority over scientific or social interests (WMA 2013, art. 8). The Nuremberg Code explicitly prohibits research that is likely to result in injury, disability or death of the research subject (art. 5 and art. 10) (Shuster 1997). In the case of human research subjects, who are not capable of giving informed consent, such as children, a common requirement is that the risk of participating in the research is minimal. “Minimal risk” is often defined as those risks that are comparable to the risks of daily life.

Animal research allows and often requires procedures that result in injury, disability or death of the animals. Our scientific and social interests have, in current practice, a clear priority over the welfare, rights (if applicable at all), and interests of the animals used for research. This difference in human and animal research ethics is only justified if animals have a lower moral status than humans. The issue of moral status is however highly contested; there is no agreement on the moral status of nonhuman animals or what exactly determines it, not even whether the concept is of any use. Two things, however, are uncontroversial. First, some nonhuman animals are capable of experiencing pain.^7^ This includes probably all vertebrates and perhaps some invertebrates (e.g. cephalopods). They have, in virtue of their sentience, morally relevant interests and possibly moral rights, and therefore they have a moral status. In other words, nonhuman animals have interests, in particular welfare interests, that have to be taken into account in our moral judgments. Second, most people intuitively accord animals a lower moral status than humans. For example, most of us would think that one should save a human child from a fire rather than a dog, if only one can be saved and we had to choose between the two. A more mundane example is the fact that most of us use animal products, including in food and clothing, knowing that animals most likely suffer and surely die in their production. This does not mean that we do not care about the death and suffering of animals, but rather that human interests are generally taken to have a priority over animal interests. Most of us would presumably agree that it is wrong to torture an animal for fun, but that we can use animals (and take their lives) as means to our own ends, if that use has reasonable benefits (which does not include sadistic pleasure). I am not arguing that current social norms or attitudes are sufficient to justify the lower moral status of animals, but rather that moral status is a fundamental issue that needs to be further analyzed and debated. This is particularly important in the case of nonhuman primates, since animals with higher cognitive capacities may have stronger welfare interests in virtue of having a stronger sense of self.

In Europe, at least, animal research is, by law, only to be approved if the researchers make a reasonable case that the expected benefits justify the harms caused to the animals. In that respect, animal research ethics overlaps here with human research ethics. The difference is, as noted above, that human research has the constraint that fundamental rights, welfare, and interests of human research subjects cannot be sacrificed for scientific or social benefits. In contrast, animal research lets scientific and social benefits justify setbacks to animal welfare and interests. There are at least two significant concerns here regarding this weighing of harms and benefits. One concern is the claim that in many animal research proposals the expected benefits only justify the suffering to the animals if their interests are massively discounted (for this concern in the context of nonhuman primates see Arnason 2018b and Faria 2018). Peter Singer has argued that if we applied a principle of equal consideration of interests, much of our animal research would not be justified (Singer 1975).

The second concern is that we cannot assess benefits or harms, whether in research on nonhuman primates or more generally in animal research, in any non-arbitrary way that would allow us to meaningfully weigh one against the other.^8^ We can, however, evaluate the expected benefits and the likely harms and make an informed, moral judgment about whether it is an acceptable trade-off. This approach acknowledges the incommensurability of harms and benefits, as well as the impossibility of accurate, objective, quantifiable measurements, without making the moral judgment trivial or arbitrary. Providing a more detailed argument for this approach is outside the scope of this paper, but this case has been made extensively elsewhere in the context of research on nonhuman primates (Arnason and Clausen 2016; Nordgren 2010).

At the beginning of this section I noted that human research ethics codes generally do not specify any upper limits of risk or harm, with the exception of the case of research subjects who are not capable of giving informed consent, in particular children. In this case, research is often considered justifiable only if it poses no more than minimal risk and minimal burden to the research subject. If we want to draw on human research ethics in our discussion of animal research ethics, this is the sort of issue that may be seen to apply directly to animals, since they cannot give informed consent either (Wendler 2014). Some animal advocates argue indeed that animal research, like nontherapeutic pediatric research, is only justifiable if it poses no more than minimal risk and minimal burden to the research subjects, at least in the case of nonhuman primates (Ferdowsian and Fuentes 2014) and great apes (Gagneux et al. 2005, p. 28). Others, such as Beauchamp and Morton (2015), have argued for upper-severity limits for animal research in general, excluding all research that causes significant suffering.

In any case, it is intellectually interesting and useful to draw comparisons between the ethical requirements for research involving incompetent humans and research involving animals with regard to upper limits of risk and harm. It is also worth emphasizing, that the justification for having different limits for humans and animals, and more generally treating human and animal interests differently, relies on an argument about the moral status of both humans and animals (Wendler 2014; Walker 2016). Placing the upper limit of risk and harm in animal research at “minimal risk and minimal harm,” as equal moral status with human would suggest, would surely amount to an abolishment of most animal research, but a higher limit of severe pain or distress over prolonged time, as is the case in EU law, would have considerably less impact on biomedical research (European Parliament and the Council of the European Union 2010).

## Conclusion

The recent move to address ethical questions in animal research within the framework of human research ethics, rather than within the more traditional framework of animal ethics, has moved the focus away from the basic question of the general, ethical justifiability of animal research and towards specific issues familiar from human research ethics, such as autonomy or agency, and harms and benefits. This move to research ethics is advocated mostly by people who are committed to some sort of an equality principle with regard to the rights or interests of human and nonhuman animals, but not necessarily from a commitment to any particular moral framework. The concern with the two topics of autonomy and harm in animal research ethics is no more and no less tied to any particular moral framework than the concern with the same topics within human research ethics. The concern with autonomy leans towards a deontological framework, the concern with harms leans towards a utilitarian framework. For both topics the Beauchamp and Childress (2013) principles of biomedical ethics loom large. Still, both topics are regularly discussed within human research ethics without a commitment to any of those moral frameworks.

How far human research ethics applies to animals depends in the end significantly on the moral status accorded to animals. If animals, or some higher mammals such as nonhuman primates, are accorded the same moral status as humans, it would be difficult to avoid the conclusion that human research ethics would apply to them, giving them the same protections as human research subjects who are not competent to give informed consent. This would be the end of much of animal research as it is practiced now. Justifying current levels of risk and harm exposure in animal research depends conversely on animals having a lower moral status than humans. This difference in moral status implies not only unequal consideration of interests, but more importantly that animal welfare and interests cannot give rise to limits based on rights or dignity that trump utilitarian considerations of harms and benefits, as is the case in human research ethics. As fruitful as recent work on animal research ethics has been, its plausibility ultimately requires a defensible account of the moral status of nonhuman animals.

## Footnotes

1 Two obvious examples are Singer‘s (1975) *Animal Liberation* and Regan‘s (2004 [1983]) *The Case for Animal Rights*, who argue for the utilitarian and deontological positions, respectively, against animal research. For further elaboration of this contrast between public views and the restrictive views within animal ethics see DeGrazia‘s (1999) “The ethics of animal research: What are the prospects for agreement?”.

2 The relationship between cognitive capacities, sentience, personhood, and moral status generally is complicated and the analysis of that relationship is outside the scope of this paper. I merely acknowledge the common assumption that nonhuman primates matter more morally than other nonhuman animals, without having the cognitive capacities necessary for moral personhood. “Personhood” (in the moral sense, as opposed to legal personhood) is a technical term indicating a full moral status, for example having a moral right to life, and was often considered coextensive with humanity (Chan and Harris 2011; Tooley 2011). Nonhuman primates are certainly sentient, where sentience, the capacity to experience pain and pleasure, is understood either as a specific cognitive capacity or a combination of cognitive capacities underlying awareness and sensation. Contrary to the common assumption in regulation and praxis, lower cognitive capacities do not necessarily imply less suffering, see for example Akhtar (2011). There remains the problem of knowing animal suffering; there is no way for us humans to know what it is like to be a mouse in pain or a severely depressed monkey, just like we cannot know what it is like to be a bat (Nagel 1974). We can merely imagine what it is like to be a human in the same situation. In terms of understanding animal suffering, the best we can do is to infer from behavioral, cognitive and neurological similarities, to what extent the experience of animal suffering may be similar to human suffering.

3 An early example is Blackmore’s (1982) paper “Animal Research Ethics at the University of Southern California.”

4 In biomedical ethics, the principlism of Beauchamp and Childress (2013) grounds moral decisions on four principles: Respect for autonomy, non-maleficence, beneficence, and justice. It combines aspects of both utilitarianism and the duty-based ethics of deontological moral theories. Within research ethics, the principle of respect for autonomy requires that research subjects can make their own informed decisions about research participation, in particular by giving their informed consent before participating in research. The principle of non-maleficence formulates an obligation to “do no harm”. The principle of beneficence formulates an obligation to benefit others. The principle of justice requires that burdens and benefits are justly distributed, which includes such concerns as the just selection of research subjects.

5 Various accounts of the concept of autonomy can be found in research ethics, but at its core is the ability to act on one’s own reasons, or to act on one’s considered judgment. Compare the following passage from the Belmont Report (National Commission 1979, B1): “To show lack of respect for an autonomous agent is to repudiate that person’s considered judgments, to deny an individual the freedom to act on those considered judgments, or to withhold information necessary to make a considered judgment, when there are no compelling reasons to do so”. Nonhuman animals, and indeed some humans, are not capable of making considered judgments and cannot be shown lack of respect as autonomous agents in the sense of this passage.

6 Here I follow Diekema’s (2006, p. S9) analysis of assent in pediatrics research, where assent “requires only that the child possess the capacity to understand that the research is not being done for his or her benefit, to understand what will happen to him or her in the research project, and to agree or disagree regarding participation”. Diekema expects most children to have the capability to assent by 7 years of age and in some cases earlier.

7 For a discussion of animal pain, see for example Allen (2004) and Rollin (2011). The view that nonhuman animals cannot experience pain, sometimes incorrectly associated with Descartes (Cottingham 1978; Harrison 1992; Thomas 2006), does currently not find any defenders of note (with the often cited exception of Harrison 1991). Although Peter Carruthers admits that (some) nonhuman animals can experience pain and other suffering, he has argued that it has no moral significance because their pain is non-conscious (Carruthers 1992, p. 192).

8 This point is often made by critics of utilitarianism, see for example Regan’s “Empty Cages: Animal Rights and Vivisection” (Regan 2005, p. 79). For a spirited attempt to provide a framework for balancing harms and benefits in animal research, see Bateson’s “When to Experiment on Animals” (Bateson 1986).

## References

[CR1] Aaltola E (2011). The philosophy behind the movement: Animal studies versus animal rights. Society & Animals.

[CR2] Akhtar S, Beauchamp TL, Frey RG (2011). Animal pain and welfare: Can pain sometimes be worse for them than for us?. The Oxford handbook of animal ethics.

[CR3] Allen C (2004). Animal pain. Nôus.

[CR4] Arnason G, da Trindade GG, Woodhall A (2017). Animal research and the political theory of animal rights. Ethical and political approaches to nonhuman animal issues.

[CR5] Arnason G (2018). Human-animal parallels in clinical ethics and research ethics. American Journal of Bioethics.

[CR6] Arnason G (2018). The ethical justification for the use of non-human primates in research: The weather all report revisited. Journal of Medical Ethics.

[CR7] Arnason G, Clausen J (2016). On balance: Weighing harms and benefits in fundamental neurological research using nonhuman primates. Medicine, Health Care and Philosophy.

[CR8] Bateson P (1986). When to experiment on animals. New Scientist.

[CR9] Beauchamp TL, Arras JD, Fenton E, Kukla R (2014). The ethics of biomedical research involving animals. The Routledge companion to bioethics.

[CR10] Beauchamp TL, Childress JF (2013). Principles of biomedical ethics.

[CR11] Beauchamp TL, Ferdowsian HR, Gluck JP (2014). Rethinking the ethics of research involving nonhuman animals: Introduction. Theoretical Medicine and Bioethics.

[CR12] Beauchamp TL, Morton DB (2015). The upper limits of pain and suffering in animal research: A moral assessment of the European Union’s legislative framework. Cambridge Quarterly of Healthcare Ethics.

[CR13] Beauchamp TL, Wobber V (2014). Autonomy in chimpanzees. Theoretical Medicine and Bioethics.

[CR14] Beecher HK (1966). Ethics and clinical research. New England Journal of Medicine.

[CR15] Blackmore WM (1982). Animal research ethics at the University of Southern California. LabAnimal.

[CR16] Brody B, Garrett JR (2012). Defending animal research: An international perspective. The ethics of animal research: Exploring the controversy.

[CR17] Buller T, Shriver A, Farah M (2014). Guest editorial: Broadening the focus. Cambridge Quarterly of Healthcare Ethics.

[CR18] Carruthers P (1992). The animal issue: Moral theory in practice.

[CR19] Chan S, Harris J, Beauchamp TL, Frey RG (2011). Human animals and nonhuman persons. The Oxford Handbook of Animal Ethics.

[CR20] Choe Smith CU (2014). Confronting ethical permissibility in animal research: Rejecting a common assumption and extending a principle of justice. Theoretical Medicine and Bioethics.

[CR21] CIOMS, Council for International Organizations of Medical Sciences (2016). International ethical guidelines for health-related research involving humans.

[CR22] Cochrane A (2018). Sentientist politics: A theory of global inter-species justice.

[CR23] Cohen C, Garattini S, van Bekkum DW (1990). Animal experimentation defended. The importance of animal experimentation for safety and biomedical research.

[CR24] Cottingham J (1978). ‘A brute to the brutes?’: Descartes’ treatment of animals. Philosophy.

[CR25] DeGrazia D (1999). The ethics of animal research: What are the prospects for agreement?. Cambridge Quarterly of Healthcare Ethics.

[CR26] DeGrazia D (2006). Regarding animals: Mental life, moral status, and use in biomedical research: An introduction to the special issue. Theoretical Medicine and Bioethics.

[CR27] DeGrazia D, Beauchamp TL (2015). Guest editorial: Reassessing animal research ethics. Cambridge Quarterly of Healthcare Ethics.

[CR28] Diekema DS (2006). Conducting ethical research in pediatrics: A brief historical overview and review of pediatric regulations. The Journal of Pediatrics.

[CR29] Donaldson S, Kymlicka W (2011). Zoopolis: A political theory of animal rights.

[CR30] European Parliament and the Council of the European Union. (1986). Directive 86/609/EEC of 24 November 1986 on the approximation of laws, regulations and administrative provisions of the member states regarding the protection of animals used for experimental and other scientific purposes. *Official Journal of the European Union* L 358, 18/12. Retrieved August 28, 2019, from http://eur-lex.europa.eu/legal-content/EN/TXT/?uri=CELEX%3A31986L0609.

[CR31] European Parliament and the Council of the European Union. (2010). Directive 2010/63/EU on the protection of animals used for scientific purposes. *Official Journal of the European Union* L 276/33; adopted 2010 Sept 22. Retrieved August 28, 2019, from http://eur-lex.europa.eu/legal-content/EN/TXT/?uri=CELEX:32010L0063.

[CR32] Faria C (2018). A flimsy case for the use of non-human primates in research: A reply to Arnason. Journal of Medical Ethics.

[CR33] Fenton A (2014). Can a Chimp say “no”?. Cambridge Quarterly of Healthcare Ethics.

[CR34] Ferdowsian H, Choe C, Corbey R, Lanjouw A (2013). Extending human research protections to non-human animals. The politics of species: Reshaping our relationships with other animals.

[CR35] Ferdowsian H, Fuentes A (2014). Harms and deprivation of benefits for nonhuman primates in research. Theoretical Medicine and Bioethics.

[CR36] Ferdowsian HR, Gluck JP (2015). The ethical challenges of animal research: Honoring Henry Beecher’s approach to moral problems. Cambridge Quarterly of Healthcare Ethics.

[CR37] Francione GL (2009). Animals as persons: Essays on the abolition of animal exploitation.

[CR38] Frey RG (1997). Moral community and animal research in medicine. Ethics and Behavior.

[CR39] Gagneux P, Moore JJ, Varki A (2005). The ethics of research on great apes. Nature.

[CR40] Garner R, O’Sullivan S (2016). The political turn in animal ethics.

[CR41] Gilbert S (2012). Progress in the animal research war. Animal Research Ethics: Evolving Views and Practices, Hastings Center Report Special Report.

[CR42] Gilbert, S., Kaebnick, G. E., & Murray, T. H. (Eds). (2012). Animal research ethics: Evolving views and practices. *Hastings Center Special Report*, 42(6). Retrieved August 29, 2019, from http://animalresearch.thehastingscenter.org/special-report/.

[CR43] Harrison P (1991). Do animals feel pain?. Philosophy.

[CR44] Harrison P (1992). Descartes on animals. The Philosophical Quarterly.

[CR45] Herrmann K, Jayne K (2019). Animal experimentation: Working towards a paradigm change.

[CR46] Hursthouse R, Welchman J (2006). Applying virtue ethics to our treatment of the other animals. The practice of virtue: Classic and contemporary readings in virtue ethics.

[CR47] IOM, Institute of Medicine (2011). Chimpanzees in biomedical and behavioral research: Assessing the necessity.

[CR48] Jaworska, A., & Tannenbaum, J. (2018). The grounds of moral status. In E. N. Zalta (Ed.), *The Stanford encyclopedia of philosophy*. Retrieved August 29, 2019, from https://plato.stanford.edu/archives/spr2018/entries/grounds-moral-status/.

[CR49] Johnson J, Barnard ND (2014). Chimpanzees as vulnerable subjects in research. Theoretical Medicine and Bioethics.

[CR50] Kantin H, Wendler D (2015). Is there a role for assent or dissent in animal research?. Cambridge Quarterly of Healthcare Ethics.

[CR51] Korsgaard CM (2018). Fellow creatures: Our obligations to the other animals.

[CR52] Merriam G, Garrett JR (2012). Virtue, vice, and vivisection. The ethics of animal research: Exploring the controversy.

[CR53] Nagel T (1974). What is it like to be a bat?. The Philosophical Review.

[CR54] National Commission for the Protection of Human Subjects of Biomedical and Behavioral Research. (1979). The Belmont report: Ethical principles and guidelines for the protection of human subjects of research. Washington, DC: Department of Health, Education and Welfare. DHEW publications (OS) 78-0013 and (OS) 78-0014. 25951677

[CR55] Nordgren A (2010). For our children: The ethics of animal experimentation in the age of genetic engineering.

[CR56] Nozick R (1974). Anarchy, state, and utopia.

[CR57] Nussbaum MC, Sunstein CR, Nussbaum MC (2004). Beyond ‘compassion and humanity’: Justice for nonhuman animals. Animal rights: Current debates and new directions.

[CR58] Pluhar EB (2006). Experimentation on humans and nonhumans. Theoretical Medicine and Bioethics.

[CR59] Rawls J (1999). A theory of justice.

[CR60] Reardon, S. (2015). NIH to retire all research chimpanzees. *Nature News*, 18 November. Retrieved August 29, 2019, from https://www.nature.com/news/nih-to-retire-all-research-chimpanzees-1.18817.

[CR61] Regan, T. (2004 [1983]). The case for animal rights. Berkeley, CA: University of California Press.

[CR62] Regan T, Cohen AI, Wellman CH (2005). Empty cages: Animal rights and vivisection. Contemporary debates in applied ethics.

[CR63] Rollin BE (2011). Animal pain: What it is and why it matters. The Journal of Ethics.

[CR64] Russell WS, Burch RL (1959). The principles of humane experimental technique.

[CR65] Shuster E (1997). Fifty years later: The significance of the Nuremberg Code. New England Journal of Medicine.

[CR66] Singer P (1975). Animal liberation.

[CR67] Singer P (1979). Practical ethics.

[CR68] Tannenbaum J, Rowan AN (1985). Rethinking the morality of animal research. Hastings Center Report.

[CR69] Thomas J (2006). Does Descartes deny consciousness to animals?. Ratio.

[CR70] Tooley M, Beauchamp TL, Frey RG (2011). Are nonhuman animals persons?. The Oxford handbook of animal ethics.

[CR71] UK Home Office (2014). Annual statistics of scientific procedures on living animals; Great Britain 2013.

[CR72] US Department of Health and Human Services. (2018). 45 CFR 46. Code of federal regulations. Title 45. Retrieved August 29, 2019, from https://www.hhs.gov/ohrp/regulations-and-policy/regulations/45-cfr-46/index.html.

[CR73] Walker RL (2006). Human and animal subjects of research: The moral significance of respect versus welfare. Theoretical Medicine and Bioethics.

[CR74] Walker RL (2016). Beyond primates: Research protections and animal moral value. Hastings Center Report.

[CR75] Walker RL (2018). Virtue, vice, and “voracious” science: How should we approach the ethics of primate research?. Perspectives in Biology and Medicine.

[CR76] Weatherall D (2006). The use of nonhuman primates in research.

[CR77] Wendler D (2014). Should protections for research with humans who cannot consent apply to research with nonhuman primates?. Theoretical Medicine and Bioethics.

[CR78] WMA, World Medical Association. (2013). *Declaration of Helsinki. Ethical principles for medical research involving human subjects*. Retrieved August 29, 2019, from https://www.wma.net/policies-post/wma-declaration-of-helsinki-ethical-principles-for-medical-research-involving-human-subjects/.

